# Surface energetics to assess influence of biomass-type and biomass–adsorbent interactions in expanded beds

**DOI:** 10.1186/s40643-021-00382-6

**Published:** 2021-04-13

**Authors:** Vikas Yelemane, Martin Kangwa, Roy N. Dsouza, Marcelo Fernández-Lahore

**Affiliations:** Downstream Bioprocessing Laboratory, School of Engineering and Science, Jacobs University, Campus Ring 1, 28759 Bremen, Germany

**Keywords:** Biofouling, Cell adhesion, Surface properties, XDLVO theory

## Abstract

In integrated bioprocessing applications, expanded bed adsorption (EBA) chromatography presents an opportunity to harvest biomolecules directly from the crude feedstock. However, unfavorable biomass interactions with adsorbent usually leads to fouling, which reduces its protein binding capacity as it alters column hydrodynamics and binding site availability. In this work, a detailed study on biomass adhesion behavior of four different industrially relevant microorganisms on 26 different, most commonly occurring adsorbent surfaces with varying degrees of surface energy and surface charge has been conducted. The results showed the derivation of a relative “stickiness” factor for every microorganism, which further classifies each organism based on their general degree of adhesion to surfaces with respect to one another. The obtained results can help to better understand the effect of biomass homogenization on biomass–adsorbent interactions in EBA. The data of surface energy and charge for the surfaces investigated in this work can be used to calculate the stickiness factor of other microorganisms of interest and may assist in the development of novel adsorbent materials for EBA chromatography.

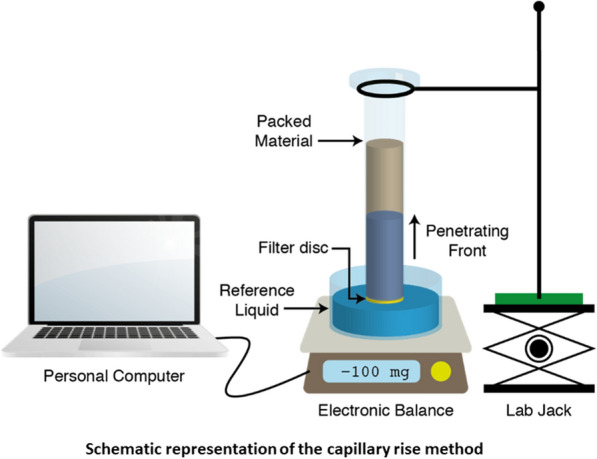

## Introduction

Interfacial interaction of a microorganism with its surrounding can happen in various ways. It can either be reversible or non-reversible, and the process usually advances in multiple stages. In 1674, Antonie van Leuwenhoek reported on the biofilm he observed in a primitive microscope from samples scraped from his tooth surface, and called them “animalcules” (little animals) (Costerton 1999), presenting the first known study of oral biofilms. In general, a biofilm micro-cluster consist of approximately 10–15% of cells and up to 75–90% of extracellular polymeric substrate matrix, where numbers may vary depending on the species involved (Costerton 1987). The development and accumulation of biofilm on surfaces may bring undesirable consequences in process environment such as in chromatography where it may lead to adsorbent fouling.

Fouling is one of the main challenges faced by industrial bioprocesses, which still needs to be solved despite significant efforts made towards its prevention. Fouling can be defined as the adhesion of unwanted material on functional surfaces that can alter their function. The most common foulants are microorganisms, biological particles, biofilms, or extracellular polymeric substrates. For decades, microbiologists and biomedical, environmental, and chemical engineers have invested a lot of effort in understanding and devising a mechanism to control microbial adhesion. Fouling results in the deterioration of equipment and increased maintenance cost (Sanders and Sturman 2005; Lappin-Scott and Costerton 1989), particularly in expanded bed chromatography (EBA) operations where biomass interactions with the stationary phase result in poor hydrodynamics of the expanded bed column (Fernández Lahore, et al. 2009). In membrane separation processes, fouling blocks the membrane irreversibly and reduces process efficiency (Kujundzic 2010), while in paper industries, these unwanted interactions of fouling agents can cause serious and costly damages to instruments (Simões et al. 2010). On the contrary, in some cases adhesion of microorganism is crucially desired, for example, in wastewater treatment plants, as well as in bio-implantable devices, where biomass interactions are required to be maximized (Melo and Bott 1997). A summary of the effects of cell adhesion/biofilms on industrial processes is listed in Table 1.

**Table 1:** A literature survey on effect of cell adhesion in various process applications

Application area	Effects of cell adhesion	References
Water systems	Contamination of purified water. Blockage of valves and entrapment of particles in waste water pipeline	Melo and Bott (1997)
Food related	Processing equipment are prone to contamination leads to germ-infested products	Simões et al. (2010), Verran (2002)
Maritime	Reduce the speed, increase fuel consumption and maintenance cost and ultimately mechanical failure	Callow and Callow (2002)
Oil	Damage of underwater cable and piping, platform wreckage	Sanders and Sturman (2005)
Paper	Operation failure and in a loss of product quality	Flemming et al. (2013)
Health	Dental plaque, infection with contact lens	Saini et al. 2011)
Industrial bioprocessing	Fouling of filtration membranes, fouling of EBA adsorbents, increase process cost	Thommes (1998), Naz (2015)

The interactions between microorganisms and surfaces are very complex in nature and are dependent on the various physico-chemical properties of the microorganism and the surface. In early stages, the adhesive interactions are reversible, however, they slowly transform into an irreversible anchorage (Fletcher 1996). In later stages, a matrix of polymeric material secreted by cells protect and stabilize their colonies. Therefore, a quantitative understanding and mechanistic interpretation of such interactions is essential to mitigate or achieve cell adhesion.

Interfacial surface properties, such as surface hydrophobicity, hydrophilicity, charge, and roughness play a very important role in the initial stages of cell adhesion to surfaces (Fletcher 1996), and can directly influence attractive or repulsive interactions and their magnitude. A previously reported model for microbial adhesion suggests that in the early stages of attachment, relatively weak non-specific and reversible interactions dominate, which eventually advance to strong irreversible states governed by both non-specific and specific interactions (Hermansson 1999). Non-specific interaction energies are generally categorized as Lifshitz–van der Waals (LW) interactions, which are always attractive and depend upon relative surface hydrophobicity, and electrostatic (EL) interactions, which can either be attractive or repulsive depending on the surface charge. These interaction energies are described in the classical DLVO (Derjaguin, Landau, Verwey, Overbeek) theory of colloid stability (Derjaguin and Landau 1941; Verwey and Overbeek 1948). In the classical DLVO approach, both interacting surfaces are assumed to be chemically inert. In case of biomass adhesion, this is not entirely true, since non-covalent interactions, such as hydrogen bonds, are also involved in the microbial adhesion mechanism. To correct this, Van Oss et al*.* introduced a short-range Lewis acid–base (AB) interaction term to account for such effects when considering proximity of microbial and adsorbent surfaces. This modified theory is termed as extended DLVO (xDLVO) theory (Oss et al. 1986, 1995; Bayoudh 2009), where surface energetic calculations of biomass and adsorbent surfaces could be used to identify conditions that are favorable or unfavorable for adhesion. The net interaction energy between cell–substrate as a function of their mutual separation (*l*) is calculated as the cumulative sum of Lifshitz–van der Waals (LW), acid–base (AB), as well as electrostatic (EL) energies (Eq. 1) (Bayoudh 2009; Meinders et al. 1995; Truesdail 1998; Vennapusa 2008a). These calculations rest upon several physico-chemical parameters which can be experimentally determined for all interacting surfaces as well as their interstitial media. Firstly, contact angles of at least three diagnostic liquids made with each surface can be used to obtain LW and AB interactions terms using the Young–Dupré equation in combination with the respective energy relations of xDLVO theory (Oss 1995; Absolom 1983). Secondly, the electrostatic behavior of each surface can be approximated via their measured zeta potentials in the medium where interactions would take place, yielding the EL interaction term in the xDLVO model (Vanoss 1993; Chen 2011). The additional AB interaction term appending classical DLVO theory has been shown to explain some of the inconsistencies between classical DLVO predictions and experimental observations (Oss et al. 1988; Oss 2006). Extended DLVO calculations have been validated in a wide variety of colloidal applications (Fernández Lahore et al. 2009; Oss 2006; Vilinska and Rao 2011; Fu et al. 2015; Kakarla 2016; Vennapusa 2008b), and has helped in the understanding of cell–substrate or cell–cell interactions.

The aim of this work was to create a “fingerprinting” method to assess cell adhesion to a variety of surfaces and to provide a general argument on cell-type-dependent adhesion. In this study, the interaction of more than 26 different surfaces with four different cells types has been investigated. Furthermore, the interactions of cell debris with the surfaces were studied. Consequently, a “stickiness” factor, which qualitatively indicates the adhesive nature of a cell type, was determined.

## Materials and methods

### Chemicals and reagents

The following polymeric beads were purchased and used in this study: cellulose from Perloza S.r.o (Czech Republic); Sepharose agarose, sephacryl, dextran from GE Healthcare (Germany); Fastline agarose from DSM biologics (The Netherlands); polyacrylamide, ceramic hydroxyapatite I and II, ceramic flouroapatite I and II from Bio-Rad (Germany); polyvinylpyrrolidone, Supelite™ DAX-8, poly(styrene-co-divinylbenzene) from Sigma-Aldrich (Germany); poly(phenol–formaldehyde), poly(ethyl methacrylate) from Polysciences (Germany); Toyopearl HW 65 from TOSOH Bioscience (Germany); and geniaLab (Germany) provided beads of agar, alginate and chitosan. Carrageenan was obtained from Carl Roth (Germany). α-Bromonaphthalene (99% purity) and formamide (99.5% purity) were obtained from Fluka, Buchs, Switzerland. Water was ultrapure quality. All other chemicals used in the buffer preparation were of analytical grade and obtained from Applichem (Germany). The goniometric system (OCA 20) was obtained from Data- Physics Instruments (Germany). Zeta potential measurements were measured using a ZetaSizer Nano ZS from Malvern Instruments (UK). A weighing balance CPA423S connected to computer via RS-232 serial connector and Sartocollect 1.0 was used to control and record the data was obtained from Sartorius AG (Germany).

### Types of biomass use

Four different types of cells with different physico-chemical characteristics were selected for the study: (a) *Escherichia coli*, (b) *Bacillus subtilis*, (c) *Saccharomyces cerevisiae*, and (d) *Chinese hamster ovary* (CHO) cells.

### Contact angle measurements

For the contact angle measurement, capillary method was used for bead material while sessile drop method was used for flat surfaces. Beads were incubated overnight, and cells were washed in 20 mM phosphate buffer at pH 7.4. For the sessile drop experiments, the diagnostic liquids: α-bromo naphthalene, formamide, and water were used to measure the contact angle using a goniometric system (OCA 20); the SCA 20 software was used for data acquisition and is from Data-Physics Instruments (Filderstadt, Germany). All the measurements were performed in triplicate and at least 20 contact angles per sample were measured. A comprehensive description of the methodologies used is published elsewhere (Sharma and Hanumantha Rao 2002; Vennapusa 2008c; Kakarla 2015).

### Zeta potential measurements

Zeta potential values were measured using Zetasizer Nano ZS. Beads were incubated in respective buffers for 2 h before the experiment. Zeta potential was calculated from the electrophoretic mobility data according to the Smoluchowski’s equation and all the measurements were done in triplicates. Details of methodology published elsewhere (Fernández Lahore et al. 2009; Kakarla 2015; Vennapusa and Fernandez-Lahore 2010).

### Total interaction energy calculation

The values of xDLVO free energy of interaction were calculated as the sum of LW, AB and EL components at the minimum separation distance (Oss 2006). The total interaction energy between a colloidal particle and a solid surface can be expressed according to the xDLVO approach using Eq. 1, where U^xDLVO^ is the total interaction energy in aqueous media, U^LW^ is the LW interaction term, U^AB^ is the AB interaction term, and U^EL^ is the EL interaction term:

1 $${U}_{\mathrm{mwc}}^{\mathrm{xDLVO}}\left(l\right)={U}_{\mathrm{mwc}}^{\mathrm{LW}}\left(l\right)+{U}_{\mathrm{mwc}}^{\mathrm{EL}}\left(l\right)+{U}_{\mathrm{mwc}}^{\mathrm{AB}}\left(l\right).$$

The subscript *m* is utilized for the chromatographic matrix, *w* refers to the watery environment, and *c* to the colloidal (cell) particle. In this work, we calculated the overall area of the interaction curve between 4 and 20 nm instead of the pocket depth as a measure of interaction. This gives a more accurate approximation of the net effect of interaction. When the interaction is attractive, the net area will be in negative range as opposed to when interactions are repulsive. A detailed explanation of underlying xDLVO calculations has been reported in our previous publications (Fernández Lahore et al. 2009; Naz 2015; Vennapusa 2008c).

## Results and discussion

Biofilm formation progresses mainly in four stages: (a) initial attachment, (b) reversible attachment, (c) maturation, (d) growth (Petrova and Sauer 2009; Svensäter and Bergenholtz 2004). These interactions are normally multimodal in nature, involving specific interactions (ligand–receptor) and non-specific interactions (hydrogen bonding, hydrophobic, van der Walls, electrostatic and other supramolecular forces). Our main objective was to derive a factor representing “stickiness” of the biomass that is qualitatively proportional to experimental results and which can only be achieved by evaluating the interaction of different types of microbes with a wide range of substrate materials and control substance.

### Selection of the representative biomass types

In this study, it was important to consider a pool of microorganisms that are commonly used in industrial biomolecule productions and that represents wider diversity in terms of surface properties. Four different cell types with distinguishable physico-chemical characteristics were selected for the study: (a) *Escherichia coli*, a Gram-negative rod-shaped bacterium with the length of approximately 1–1.5 µm; (b) *Bacillus subtilis*, a Gram-positive rod-shaped bacterium approximately 2–3 µm length; (c) *Saccharomyces cerevisiae*, a yeast, most studied eukaryotic model ovoid in shape and up to 10 µm in diameter, and (d) Chinese hamster ovary (CHO) cells, which is the most common mammalian host and is approximately 14–15 µm in diameter. The above-mentioned cell types represent the most common microbial flora encountered in cell–substrate interaction in bioprocess industry. The differential properties between these microorganisms include cell wall structure, composition, and extracellular appendages.

### Selection of the polymeric beads made of different materials

Twenty-six common polymeric materials encountered in manufacturing and bioprocess industries were physico-chemically characterized. Polysaccharides such as alginate, agar, cellulose, agarose, carrageenan, dextran, and chitosan are the most common raw materials utilized as beads in process engineering. Chemical polymers such as polyacrylamide, sephacryl, polyvinylpyrrolidone, polyphenolformaldehyde beads, Supelite™ DAX-8, poly(styrene-co-divinylbenzene), poly(ethyl methacrylate), polystyrene, Toyopearl HW-65, silica, ceramic hydroxyapatite type I and II, and ceramic fluoroapatite type I and II are widely used in various adsorption processes. Additionally, construction materials such as glass, stainless steel, hexamethyldisilazane, and polydimethylsiloxane have been included in this study. This collection of materials involves a wide range of hydrophilic, hydrophobic, and surface charge properties. For calculations, the diameter of the interacting bead made from these materials is assumed to be 200 µm.

Additionally, 4 agarose-based bead materials as benchmarks for the comparison of calculated interactions were employed. Sepharose DEAE and Q, anion exchangers which harbor positive surface charge and are known to interact strongly with microbial cells. Sepharose SP a cation exchanger harboring negative surface charge, and generally exhibits the least interaction with biomass, and phenyl-Sepharose, which possesses hydrophobic ligands, allows for the comparison of relative hydrophobicity involved in adhesive interactions.

### Contact angle measurements

Contact angles of diagnostic liquids, namely, water, formamide, and 1-bromonaphthalene with several materials were used to estimate their surface energy parameters. Nonpolar 1-bromonaphthalene solely determines the Lifshitz–van der Waals (LW) component, and polar liquids, water and formamide, determine the acid–base (AB) nature of the surfaces. Angles were calculated using the capillary rise method (Kakarla 2015) for beads and the sessile drop method (Vennapusa 2008b) for flat surfaces. Most of the chromatographic adsorbents used in protein purification are highly hydrophilic in nature, thereby making them heavily hydrated during chromatographic operations. Due to their hydrated state, it was assumed that these materials have a zero contact angle with water, which is supported by previous reports (Sharma and Hanumantha Rao 2002; Kakarla 2015; Aasim 2014). This assumption enabled us to use water as the completely wetting liquid for contact angle estimation using the Washburn equation (Washburn 1921). This method has been applied to various chromatographic adsorbents and its applicability has been validated in our previous publication (Kakarla 2015). Contact angles for 23 beads were calculated from their respective wetting kinetic slopes with water, formamide, and 1-bromonaphthalene. All the beads were equilibrated in 20 mM phosphate buffer at pH 7.4 before the measurements, which resembles a chemical environment similar to that found in process conditions. All experiments were done in triplicates and their standard deviations are reported in Table 2. Contact angle values for commercial chromatographic adsorbents Sepharose Q, DEAE, SP and phenyl, along with polymeric beads made of polysaccharides, polymers, and biomass are tabulated. The surface properties of carrageen were measured with carrageen hydrogels prepared using powder and were measured using the sessile drop method. Contact angles and zeta potential data for biomass, glass, stainless steel, polystyrene, hexamethyldisilazane, polydimethylsiloxane and silica were obtained from published literature (Comelles et al. 2010; Hedberg 2013; Carré 2007; Helms 2012). Using the Young–Dupre equation, surface energy parameters of polar liquids (water and formamide) were used to determine acid–base (AB) nature of the surfaces, whereas the apolar liquid (1-bromonaphthalene) for the Lifshitz–van der Waals (LW) component.

**Table 2:** Contact angles of for biomass, control adsorbents and polymeric beads

	**Contact angle (θ)**		
Material	**H**_**2**_**O**	**FMD**	**ABN**
Biomass			
*E. coli*^*1*^	26.8 ± 0.8	31.0 ± 1.4	44.3 ± 1.0
*B. subtilis*^*2*^	33.0 ± 2.0	45.0 ± 2.0^G^	66.0 ± 2.0^D^
*S. cerevisiae*^*3*^	15.0 ± 2.0	14.0 ± 1.0	54.0 ± 1.0
CHO^1^	26.1 ± 0.7	30.2 ± 0.2	42.3 ± 0.5
Control			
Sepharose Q	0	36.4 ± 0.3	65.3 ± 3.3
Sepharose DEAE	0	29.2 ± 0.6	55.9 ± 0.1
Sepharose SP	0	32.5 ± 0.4	56.4 ± 1.3
Sepharose phenyl	0	33.4 ± 0.5	56.6 ± 2.4
Polymeric material			
Alginate	0	43.3 ± 0.3	63.3 ± 0.4
Agar	0	42.2 ± 0.5	65.6 ± 2.6
Cellulose	0	41.2 ± 0.3	64.1 ± 1.8
Sepharose 4B	0	24.8 ± 1.1	54.2 ± 0.5
Fastline agarose	0	38.0 ± 0.1	60.5 ± 1.0
Sephacryl S 400	0	39.9 ± 0.4	65.8 ± 1.8
Carrageenan	15.3 ± 1	25.1 ± 1.5	60.7 ± 0.8
Dextran (PD 10)	0	41.6 ± 0.5	51.4 ± 0.2
Polyacrylamide	0	44.7 ± 0.7	55.1 ± 0.1
Polyvinylpyrrolidone	0	42.2 ± 0.1	44.6 ± 0.2
Phenolic beads	0	38.7 ± 0.5	41.4 ± 0.2
Supelite™ DAX-8	0	34.3 ± 0.7	37.3 ± 0.1
Poly(styrene-co-divinylbenzene)	0	35.3 ± 0.5	37.2 ± 0.6
Poly(ethyl methacrylate)	0	47.1 ± 0.3	53.7 ± 0.4
Chitosan	0	35.8 ± 0.5	37.7 ± 0.6
Glass^4^	35.0 ± 6.0	36.0 ± 5.0	52.0 ± 1.0^D^
Stainless steel^5^	81.2 ± 0.9	60.0 ± 1.1	23.4 ± 0.5
Polystyrene^6^	85.4 ± 0.7	65.3 ± 1.6	41.4 ± 1.5^D^
Toyopearl HW-65	0	22.48 ± 1.9	65.8 ± 1.4
Hexamethyldisilazane (HMDS)^7^	90.8 ± 1.5	72.9 ± 2.0	55.3 ± 0.8
Polydimethylsiloxane (PDMS) ^4^	107.0 ± 5.0	97.0 ± 2.0	67.0 ± 2.0^D^
Silica^7^	4.7 ± 1.5	9.5 ± 2.0	17.4 ± 2.4
Ceramic hydroxyapatite type 1	0	51.22 ± 0.3	53.2 ± 0.8
Ceramic hydroxyapatite type 2	0	43.7 ± 1.6	56.6 ± 0.2
Ceramic fluoroapatite type 1	0	44.8 ± 0.5	57.9 ± 1.9
Ceramic fluoroapatite type 2	0	40.9 ± 0.6	61.7 ± 0.6

Superscripted numbers on the colloids refers to the source of the data, ^1^ is from (Kakarla 2015); ^2^ is from (Li and Logan 2004); ^3^ is from (Vennapusa 2008b); ^4^ is from (Comelles, et al. 2010); ^5^ is from (Hedberg 2013); ^6^ is from (Carré 2007); ^7^ is from (Helms 2012). ^G^ and ^D^ on contact angles refers to angles of diagnostic liquids glycerol and diiodomethane, respectively

According to van Oss (Oss 2006), the hydrophilic/hydrophobic nature of a certain material can be defined in terms of the variation of the free energy of interaction (ΔG_sws_) between two surfaces (*s*) of that material immersed in water (*w*). The values of ΔG_sws_ were mostly positive for all the materials used except stainless steel, polystyrene, HMDS and PDMS. Beads having ΔG_sws_ >  + 25 mJ m^−2^ indicate their hydrophilic nature (Vennapusa 2008c). Lower γ^LW^ values were obtained for Toyopearl HW-65 and Sephacryl S 400 beads when compared to agarose (fastline and Sepharose). From the data obtained, polymers can be arranged based on polar character in the following sequence: Toyopearl HW-65 and Carrageenan (5.5—4.4) > agarose (2.7–1.5) > ceramic hydroxyapatite type 1 (0). Biomass with ΔG_sws_ > 0 describes hydrophilic cell surfaces, which have a lower tendency to form aggregates. All of the surface energy parameter values are listed in Table 3.

**Table 3:** Surface energy parameters for biomass, control adsorbents and polymeric beads

	Surface energy parameters (mJ m^-2^)					
Material	γ^LW^	γ^+^	γ^**−**^	γ^AB^	γ_S_^**Total**^	ΔG_sws_
Biomass						
*E. coli*	32.70	1.40	51.80	17.03	49.73	31.01
*B. subtilis*	44.00	0.10	59.20	4.87	48.87	42.35
*S. cerevisiae*	27.90	4.40	51.50	30.11	58.01	24.36
CHO	33.60	1.20	52.10	15.81	49.41	30.92
Control						
Sepharose Q	22.30	2.56	71.39	27.06	49.36	46.90
Sepharose DEAE	27.02	2.36	65.20	24.81	51.82	41.95
Sepharose SP	26.80	1.90	68.11	22.75	49.55	46.52
Sepharose Phenyl	26.69	1.63	69.96	21.34	48.03	49.53
Polymeric material						
Alginate	23.32	1.05	79.69	18.30	41.61	62.37
Agar	22.18	1.50	78.09	21.65	43.83	57.94
Cellulose	22.93	1.48	76.97	21.33	44.26	57.08
Sepharose 4B	27.88	2.78	61.92	26.22	54.10	37.41
Fastline Agarose	24.69	1.57	73.46	21.45	46.14	53.32
Sephacryl S 400	22.06	1.96	75.21	24.30	46.37	52.89
Carrageenan	24.64	4.21	56.55	30.84	55.48	29.45
Dextran (PD 10)	29.23	0.31	78.45	9.92	39.16	67.29
Polyacrylamide	27.50	0.25	82.18	9.12	36.62	72.37
Polyvinylpyrrolidone	32.55	0.05	79.54	3.98	36.53	72.55
Phenolic beads	33.99	0.11	75.55	5.81	39.80	66.01
Supelite™ DAX-8	35.75	0.21	71.06	7.73	43.48	58.63
Poly(styrene-co-divinylbenzene)	35.79	0.15	72.10	6.68	42.47	60.65
Poly(ethyl methacrylate)	28.13	0.07	85.64	4.78	32.91	79.78
Chitosan	35.61	0.14	72.57	6.47	42.08	61.43
Glass	33.15	0.98	46.20	13.49	46.64	25.98
Stainless steel	40.94	0.00	5.53	0.32	41.26	− 59.75
Polystyrene	38.90	0.00	2.83	0.00	38.90	− 72.94
Toyopearl HW-65	22.02	5.56	59.59	36.41	58.43	28.74
Hexamethyldisilazane (HMDS)	27.34	0.09	3.89	1.19	28.53	− 59.09
Polydimethylsiloxane (PDMS)	24.56	0.00	0.03	0.00	24.56	− 98.58
Silica	42.39	0.81	55.84	13.45	55.84	33.43
Ceramic Hydroxyapatite Type 1	28.34	0.00	89.71	0.00	28.34	88.46
Ceramic Hydroxyapatite Type 2	26.65	0.44	80.69	11.87	38.52	68.56
Ceramic Fluoroapatite Type 1	26.04	0.41	82.10	11.55	37.59	70.42
Ceramic Fluoroapatite Type 2	24.12	1.24	76.75	19.53	43.65	58.30

### Zeta potential measurement

Zeta potential plays a key role in the study of biomass–adsorbent interactions (Lin 2003; Lin et al. 2006). Zeta potential measurements provide the surface charge of a particle or surface as a function of solution chemistry, i.e., ionic strength, electrolyte composition, and pH. Zeta potentials were measured in phosphate buffer with varying salt (NaCl) concentration (0 mM, 25 mM, 50 mM, 100 mM), which enabled its calculation over an entire range of ionic strengths using empirical scaling functions (Kirby and Hasselbrink 2004). Table 4 summarizes zeta potential values for all materials in this study. Zeta potentials were obtained in two conditions, namely, (a) binding condition (20 mM phosphate buffer pH 7.4), and (b) elution condition (20 mM phosphate buffer pH 7.4 and 150 mM NaCl). In biomass, *B. subtilis* showed slightly higher negative charge compared to others. In control experiments, Sepharose Q and DEAE showed positive charge on the surface, whereas phenyl was slightly negative and SP was having higher negative change for control materials. Polymeric materials showed a wider range of zeta potential values ranging from positive to negative.

**Table 4:** Zeta potential values for biomass, control adsorbents and polymeric beads

	Zeta potential mV	
	Binding condition	Elution condition
Biomass		
*E. coli*^1^	− 29	− 23
*B. subtilis*^2^	− 40	− 24
*S. cerevisiae*^3^	− 20	− 11
CHO^1^	− 18	− 11
Control		
Sepharose Q	27	13
Sepharose DEAE	19	10
Sepharose SP	− 23	− 12
Sepharose phenyl	− 2	− 1
Polymeric material		
Alginate	− 21	− 14
Agar	− 26	− 16
Cellulose	− 5	− 2
Sepharose 4B	− 2	0
Fastline agarose	− 7	− 3
Sephacryl S 400	− 1	− 1
Carrageenan	− 26	− 14
Dextran (PD 10)	0	1
Polyacrylamide	− 2	2
Polyvinylpyrrolidone	− 1	0
Phenolic beads	− 29	− 17
Supelite™ DAX-8	− 8	− 3
Poly(styrene-co-divinylbenzene)	− 15	− 7
Poly(ethyl methacrylate)	− 27	− 15
Chitosan	2	1
Glass^4^	− 24	− 7
Stainless steel^5^	− 38	− 15
Polystyrene^6^	− 24	− 13
Toyopearl HW-65	− 7	0
Hexamethyldisilazane (HMDS)^7^	0	0
Polydimethylsiloxane (PDMS)^4^	− 64	11
Silica^7^	− 26	− 16
Ceramic hydroxyapatite type 1	− 37	− 17
Ceramic hydroxyapatite type 2	− 50	− 19
Ceramic fluoroapatite type 1	− 46	− 18
Ceramic fluoroapatite type 2	− 46	− 20

Superscripted numbers on the colloids refers to the source of the data, ^1^ is from (Kakarla 2015); ^2^ is from (Li and Logan 2004); ^3^ is from (Vennapusa 2008b); ^4^ is from (Comelles, et al. 2010); ^5^ is from (Hedberg 2013); ^6^ is from (Carré 2007); ^7^ is from (Helms 2012). Rest are own measurements. The binding buffer is 20 mM phosphate buffer with 7.4. Elution condition is 20 mM phosphate buffer with 7.4 + 150 mM NaCl

### Interaction energy calculation

Researchers, including our group, have shown that theoretical calculations based on xDLVO theory can predict the interactions that are in agreement with experiments in various applications, such as, chromatography (Vennapusa 2008b), biofilm formation (Nguyen 2016), nanoparticle transport (Mikelonis et al. 2016) and others in aqueous media based on surface chemistry principles. Total interaction energies between biomass and adsorbent at corresponding solution conditions were calculated as a function of spatial separation between them, using surface energy parameters from contact angle and zeta potential measurements. All the calculation were implemented according to a sphere-to-plate geometry (Vennapusa 2008c). This is because in comparison with the size of the cell, the size of the adsorbent is very large (Vennapusa 2008b). These calculations will generate interaction energy *vs.* distance profiles, which will determine whether the nature of the interaction is positive or negative.

**Fig. 1:**
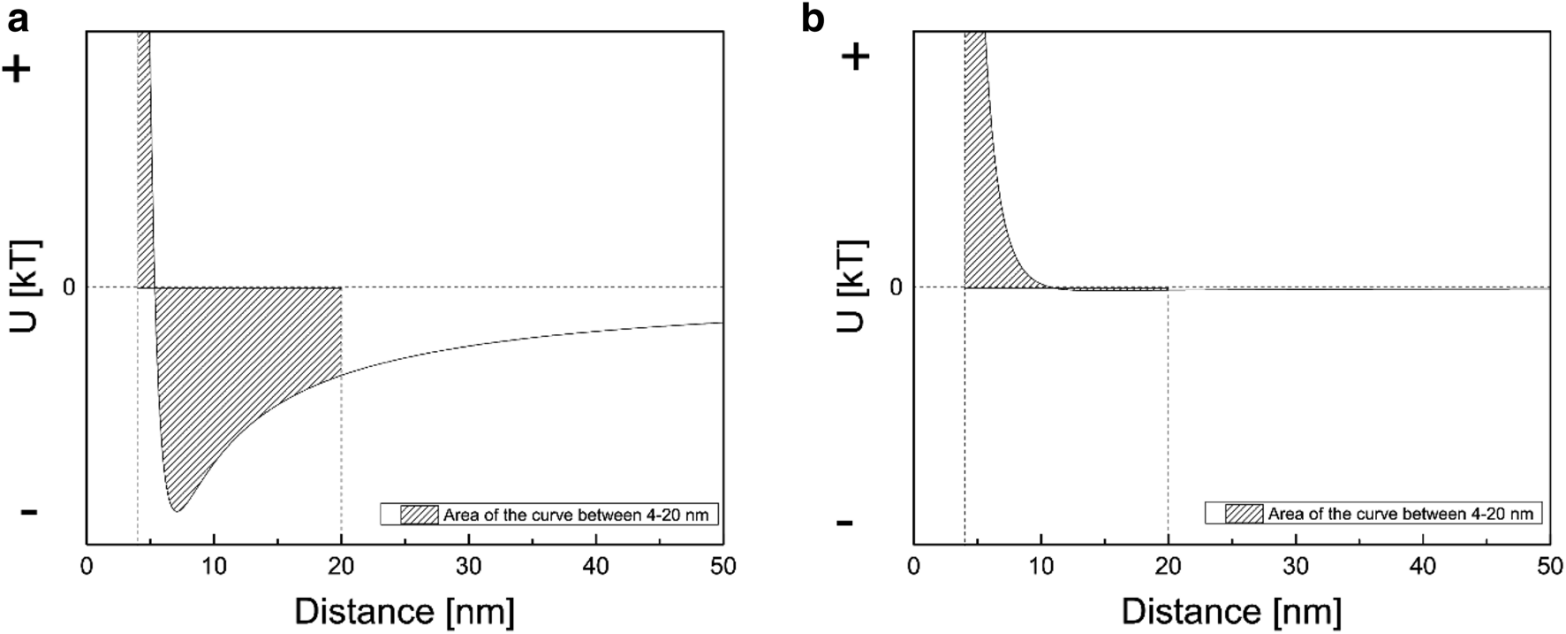
Calculating area of the interaction energy curve **a** for attractive surfaces, **b** for repulsive surfaces

**Fig. 2:**
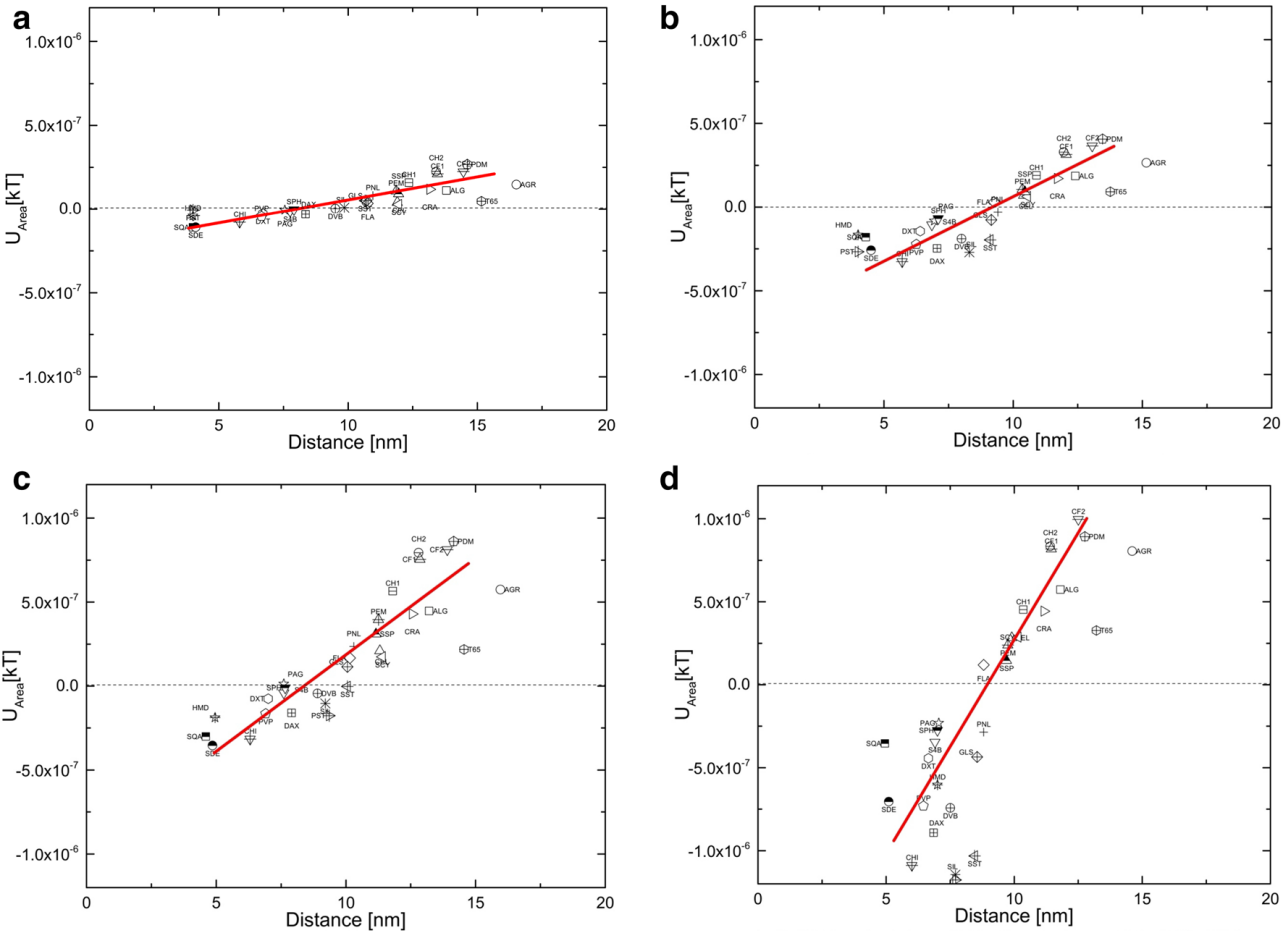
The interaction energy Vs distance profiles of biomass as function of distance for **a** *E. Coli*, **b** *B. subtilis,* **c** *S. cerevisiae* and **d** CHO cells interaction with various polymeric beads in 20 mM phosphate buffer at pH 7.4, trend line on the graph is for representation of the trend

When organisms approach the surface of an adsorbent, the physical interaction forces that are believed to provide the stimulus in initial adhesion of the organisms are: (a) hydrophobic interactions from 0.5 to 2 nm, where attached water on the surface poses a potential barrier for specific interaction; (b) repulsive and attractive electrostatic interactions from 2–10 nm, which are generally repulsive beyond 10 nm, and finally (c) long-range Lifshitz–van der Waals interactions, which commonly occur at less than 50 nm of separation (Fletcher 1996). Another very important class of interactions are ligand–receptor interactions, which are very specific physico-chemical forces between biological molecules and surfaces. Ligand–receptor interactions are strong and act within a short-range of distances less than 1 nm (Helm et al. 1991). These interactions cannot be accounted for within xDLVO theory. In current experiment, instead of the pocket depth as a measure of interaction, we calculated the overall area of the interaction curve between 4 and 20 nm. When the interaction is attractive the net area will be negative, whereas when interaction is repulsive, the resulting area will be positive (Fig. 1). While the area of the interaction energy curve provides a net estimation of the total interaction between the biomass and adsorbent, it was careful to consider the distance at which secondary minima occurs, as it is a measure of the intensity of interaction. By plotting these two values for each adsorbent with a particular cell type, it was possible to obtain from the slope of such a scatter diagram a general tendency for the cell to adhere upon different surfaces (Fig. 2). We term these slope values as the “stickiness” factor of the cell type. Interestingly, the “stickiness” factor of a cell increases qualitatively with its complexity, where *E. coli* exhibits a relatively lower stickiness as compared to CHO cells.

“Stickiness” factor plots for different types of the cells are presented in Fig. 2. *E. coli* cells showed the least interaction with the polymers, followed by *B. subtilis* then *S. cerevisiae* and CHO cells, which showed larger interactions, both for repulsive as well as attractive. “Stickiness” factor values were 2.21 × 10^–08^, 6.33 × 10^–08^, 1.06 × 10^–07^ and 2.54 × 10^–07^, for *E. coli, B. subtilis, S. cerevisiae* and CHO cells, respectively. This interaction trend is in direct agreement with biomass interaction problems encountered in process conditions and researchers have already reported that *E. coli* generally exhibited lower interaction when compared to yeast cell (Anand, et al. 2007; Balasundaram and Harrison 2005,2008; Balasundaram 2008). Moreover, Fig. 2 clearly exemplifies and confirms the intuitive notion that as the cell complexity of the biomass increases (*E. coli* to CHO), the intensity of the interaction also increases.

**Fig. 3:**
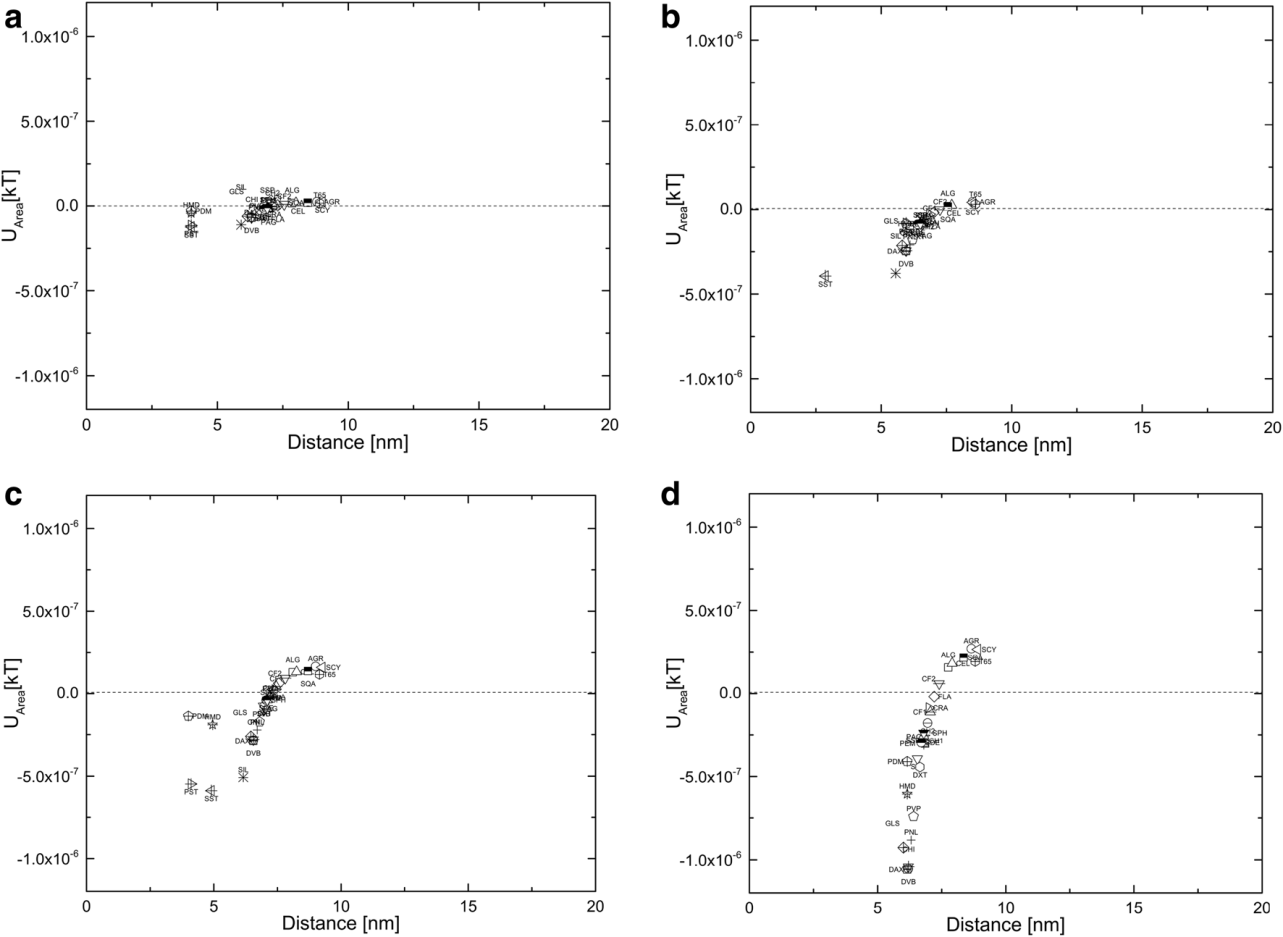
The interaction energy Vs distance profiles of biomass as function of distance for **a** *E. Coli*, **b** *B. subtilis,* **c** *S. cerevisiae* and **d** CHO cells interaction with various polymeric beads in 20 mM phosphate buffer at pH 7.4 with 150 mM NaCl

The effect of the salt concentration was on biomass interactions was also investigated by conducting the calculations in 20 mM phosphate buffer at pH 7.4 with 150 mM NaCl. High salt conditions are known to suppress electrostatic interactions due to screening, thereby shifting the mode of interaction to mainly LW interactions, which act on at much shorter distances. As a result, the secondary minima of the interaction energy curves are shifted to much smaller distances for all biomass/polymer pairs. Furthermore, since LW interactions are always attractive, repulsive interactions that originated due to electrostatic effects are severely curtailed in these conditions, we expect to observe an apparent increase in biomass “stickiness” factors. Confirming this effect, the “stickiness” factor values for *E. coli, B. subtilis, S. cerevisiae* and CHO cells were 2.4 × 10^–08^, 9.54 × 10^–08^, 1.29 × 10^–07^ and 4.75 × 10^–07^, respectively, which are slightly higher than those measured in low salt conditions (Fig. 3). Consequently, the interactions of polymers with *E. coli* appears to be relatively independent of the nature of the polymer, which is represented by the formation of a cluster in the graph. A similar trend is observed for *B. subtilis* and *S. cerevisiae*. However, CHO interactions were quite dominant even in the presence of salt. It was observed that most of the polysaccharide-based polymers were repellant in nature except for chitosan. Hydrophobic polymers showed less interaction with *E. coli* and the complexity increased as they showed increase in interaction and also their interaction increased in presence of salt.

**Fig. 4:**
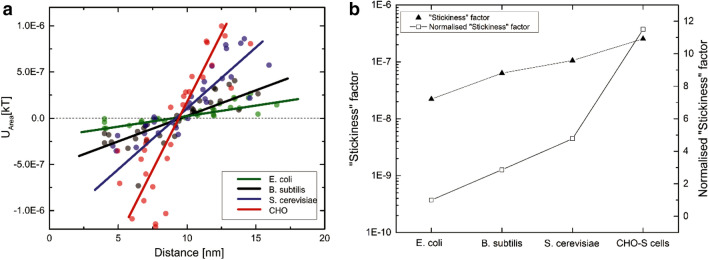
**a** A combined interaction energy Vs distance plot for *E. coli*, *B. subtilis*, *S. cerevisiae* and CHO cells as function of distance in 20 mM phosphate buffer at pH 7.4; **b** absolute values of “Stickiness” factor compared with normalized one

Figure 4 presents a compiled version of the above discussed results plotted for easy one-to-one comparison where *E. coli* shows the least interaction with the polymers forming the lowest stickiness factor and keep on increasing till CHO cells. To evaluate how stickier one type of cells compared to another, normalized “Stickiness” factor was calculated. To calculate the normalized “Stickiness” factor *E. coli* is considered as the reference point, its normalized “Stickiness” factor values are considered to be one and the ratios calculated with other cell types. The main rationale to choose *E. coli* as reference point is from the previous published literature where it was known that they are compatible with most type of EBA adsorbents and show least interaction compared to other cell types. For interaction in 20 mM phosphate buffer at pH 7.4, normalized “Stickiness” factor values showed *B. subtilis* are 2.86 times, *S. cerevisiae* are 4.77 times and CHO cells are 11.49 times stickier in comparison with *E. coli* cells. Interaction in 20 mM phosphate buffer at pH 7.4 with 150 mM NaCl, normalized “Stickiness” factor values showed trends similar to buffer condition without salt, however absolute values were different. *B. subtilis* are 3.98 times, *S. cerevisiae* are 5.38 times and CHO cells are 19.79 times stickier in comparison to *E. coli* cells.

### Effect of size on interaction and in terms of “Stickiness” factor

**Fig. 5:**
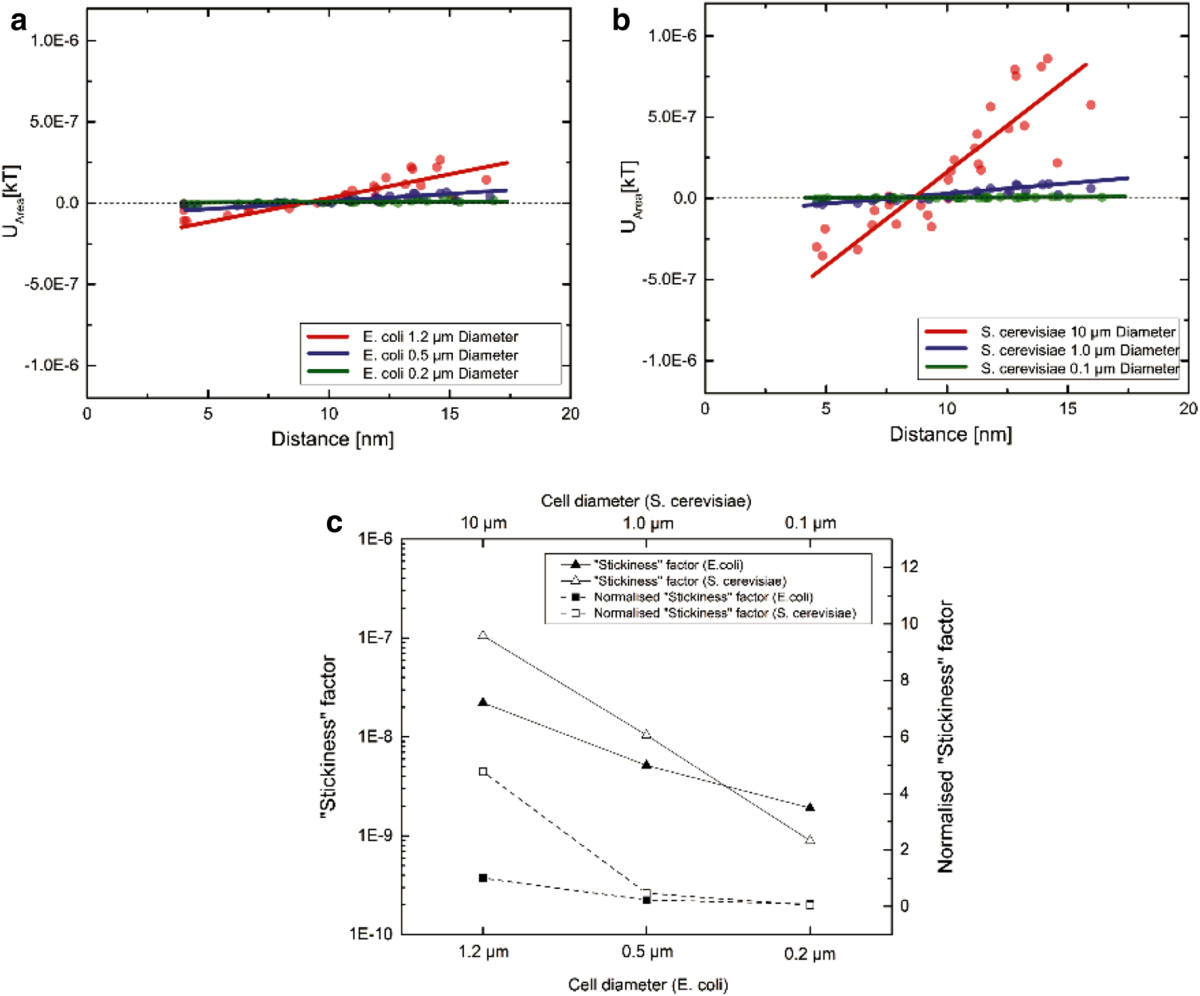
Effect of particle size on interaction surface **a** with *E. coli, b* with yeast interaction is presented; **c** comparison of “Stickiness” factor change with particle size and cell type

The effect of particle size on interaction has been investigated using model biomass and its effect on biomass interaction can be explained using the “stickiness” factor (Fig. 5). The reduction of biomass interaction problem in expanded bed systems upon cell lysis has been well documented in the literature (Anand et al. 2007; Balasundaram and Harrison 2005,2008; Balasundaram 2008). In this study, we used data from one such report (Balasundaram and Harrison 2005), where homogenized feedstock, typical for *E. coli* applications in the industry, used in an EBA experiment and compare it with our calculations. Balasundaram et al. studied the extent of disruption of *E. coli* over a wide range and its effect on the bed expansion and adsorption (Anand, et al. 2007; Balasundaram and Harrison 2005). For our calculations, we assumed that after several passes of a cell suspension through a high-pressure homogenizer, cell particle diameters are reduced to between 0.2 and 0.5 µm compared to a whole-cell diameter of 1.2 µm. After performing xDLVO calculations, the “stickiness” factor dropped from 2.21 × 10^–08^ to 5.15 × 10^–09^ at 0.5 µm then further to 1.91 × 10^–09^ at 0.2 µm particle diameters. The normalized “stickiness” factor dropped almost ten times for a concomitant sixfold reduction in particle size. These findings demonstrate that *E. coli* particle sizes indeed influence the interaction with adsorbents significantly.

The influence of the extent of disruption of baker’s yeast on protein adsorption in expanded beds has also been conducted by Balasundaram et al. (Balasundaram and Harrison 2008). They reported that an increase in the intensity of disruption resulted in an increase in the dynamic binding capacity of both the total soluble protein and α-glucosidase in expanded bed adsorption chromatography (Balasundaram 2008; Balasundaram and Harrison 2008). In this case, the “stickiness” factor for whole cells with 10 µm diameters was found to be 1.06 × 10^–07^, after a tenfold reduction in particle size it dropped to 1.04 × 10^–08^, and finally with a 0.1-µm diameter it dropped to 8.95 × 10^–10^. Normalized “Stickiness” factors were 4.77, 0.47 and 0.04, respectively, for 10, 1 and 0.1 µm, and were in direct proportion to each other. These results demonstrate the degree of biomass–adsorbent interactions is in direct proportion to the size of the biomass particle as well as its “stickiness” factor and that these experimental observations can be logically explained using xDLVO theory.

## Conclusion

In this work, a “stickiness” factor was derived based on the interactions of several types of cells with a library of adsorbents having diverse surface properties, which can help in the calculation of the stickiness level of any physico-chemically characterized microorganism. This “stickiness” factor has been shown to approximate the tendency of a microorganism to interact with surfaces in general. It was also found that interactions were stronger at lower salt concentrations and decreases as salt concentrations are increased. Cell particle sizes also were shown to play an important role in determining the intensity of interaction. We validated the universal applicability of “Stickiness” factors within a single cell type having different cell particle size and these results corroborated earlier published experimental findings. In summary, this work could help compare the biomass–adsorbent interactions for different biomass types as well as different biomass particle sizes. Furthermore, the data acquired in this work could potentially aid the development of next-generation EBA adsorbents having lesser biomass interaction.

## Data Availability

All data are fully available without restriction.

## Abbreviations

*B. subtilis*: *Bacillus subtilis*
CHO: Chinese hamster ovary
DEAE: Diethylaminoethyl
DLVO: Derjaguin, Landau, Verwey, and Overbeek theory
DSP: Downstream processing
*E. coli*: *Escherichia coli*, (B)
EBA: Expanded bed adsorption
EL: Electrostatic
LW: Lifshitz–Van der Waals
*Saccharomyces cerevisiae*: *S. cerevisiae*
xDLVO: Extended Derjaguin, Landau, Verwey, Overbeek
